# *Ab initio* description of oxygen vacancies in epitaxially strained $\hbox {SrTiO}_{{3}}$ at finite temperatures

**DOI:** 10.1038/s41598-021-91018-4

**Published:** 2021-06-01

**Authors:** Zizhen Zhou, Dewei Chu, Claudio Cazorla

**Affiliations:** 1 School of Materials Science and Engineering, UNSW Australia, Sydney, NSW 2052 Australia; 2 Departament de Física, Universitat Politècnica de Catalunya, Campus Nord B4-B5, 08034 Barcelona, Spain

**Keywords:** Materials for energy and catalysis, Nanoscale materials, Theory and computation

## Abstract

Epitaxially grown $\hbox {SrTiO}_{{3}}$ (STO) thin films are material enablers for a number of critical energy-conversion and information-storage technologies like electrochemical electrode coatings, solid oxide fuel cells and random access memories. Oxygen vacancies (${\mathrm{V}_{{\mathrm{O}}}}$), on the other hand, are key defects to understand and tailor many of the unique functionalities realized in oxide perovskite thin films. Here, we present a comprehensive and technically sound *ab initio* description of ${\mathrm{V}_{{\mathrm{O}}}}$ in epitaxially strained (001) STO thin films. The novelty of our first-principles study lies in the incorporation of lattice thermal excitations on the formation energy and diffusion properties of ${\mathrm{V}_{{\mathrm{O}}}}$ over wide epitaxial strain conditions ($-4 \le \eta \le +4$%). We found that thermal lattice excitations are necessary to obtain a satisfactory agreement between first-principles calculations and the available experimental data for the formation energy of ${\mathrm{V}_{{\mathrm{O}}}}$. Furthermore, it is shown that thermal lattice excitations noticeably affect the energy barriers for oxygen ion diffusion, which strongly depend on $\eta$ and are significantly reduced (increased) under tensile (compressive) strain. The present work demonstrates that for a realistic theoretical description of oxygen vacancies in STO thin films is necessary to consider lattice thermal excitations, thus going beyond standard zero-temperature *ab initio* approaches.

## Introduction

Crystalline defects, namely, deviations from the ideal periodic arrangement of atoms in crystals, are ubiquitous in real solids. Fortunately, the presence of crystalline defects may be desirable for enhancing the functionality of some materials^1–4^. A quintessential example of a functional type of crystalline defect is oxygen vacancies (${\mathrm{V}_{{\mathrm{O}}}}$). Oxygen vacancies, for instance, can drastically boost the catalytic activity of transition metal oxide (TMO) surfaces by providing abundant reactive sites as well as highly mobile charges^4–6^. The magnetic properties of TMO also can be altered substantially by changing their oxygen content since the exchange interactions between transition metal ions typically are sustained by O atoms^7–9^. Moreover, the presence of ${\mathrm{V}_{{\mathrm{O}}}}$ enables ionic conductivity in many perovskite-based solid solutions that are employed in modern electrochemical applications like solid oxide fuel and electrolysis cells^10,11^. Consequently, the functionality of TMO materials can be tailored and finely tuned through their stoichiometry.

**Figure 1:**
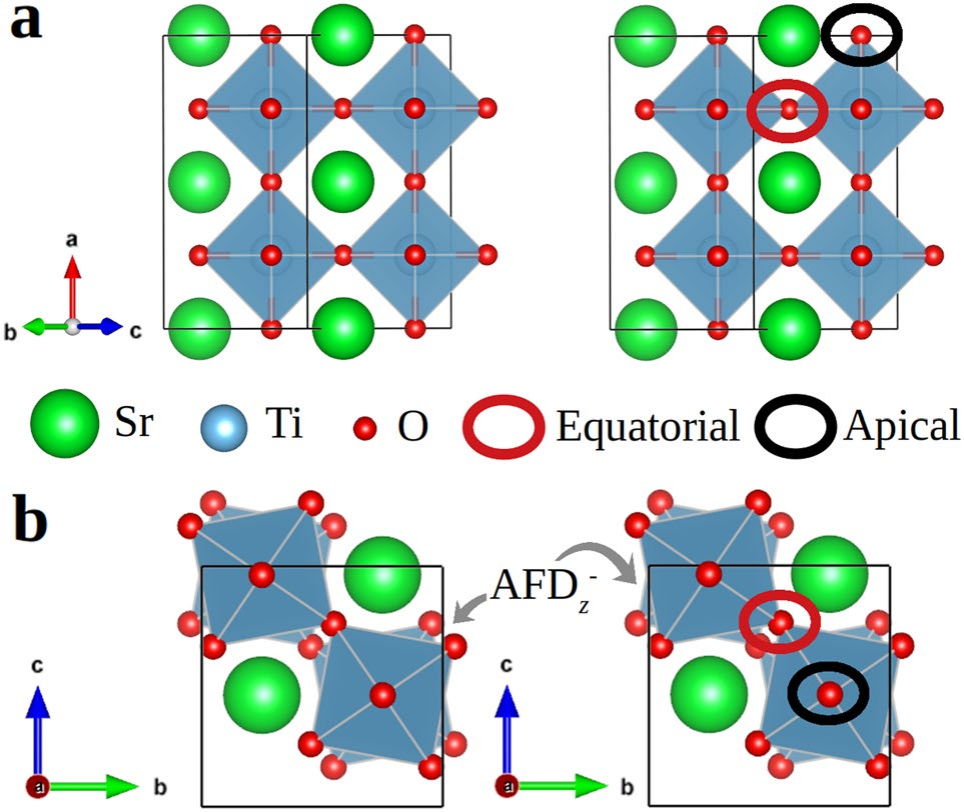
Representation of the 20-atoms simulation cell employed for the simulation of stoichiometric and epitaixally strained (001) $\hbox {SrTiO}_{{3}}$ thin films. Different views of the simulation cell are represented in (**a**) and (**b**) along with the typical antiphase out-of-plane oxygen octahedral antiferrodistortive distortions ($\hbox {AFD}_{z}^{-}$) found in (001) $\hbox {SrTiO}_{{3}}$ thin films. The generic positions of equatorial and apical oxygen ions are indicated in the figure and the Ti atoms are located inside the shaded $\hbox {O}_{{6}}$ octahedra.

Another functionality design strategy that has proved very successful for TMO materials is strain engineering^12–14^. Strain engineering consists in growing epitaxial thin films on top of substrates that present a lattice parameter mismatch, $\eta$. Either compressive or tensile biaxial stress can thus be introduced in thin films upon the condition of coherent elastic coupling with the substrate. The ferroelectric^14–18^, magnetic^8,19^, optical^20,21^ and catalytic^12,21^ properties of TMO thin films can be drastically changed by strain engineering due to the existing strong couplings between their structural and electronic degrees of freedom.

Recently, it has been realized that strain engineering also can be used to tune the formation and diffusion of oxygen vacancies in oxide thin films, and that such a combined physico-chemical approach represents a very promising technique for engineering new materials^22–25^. An illustrative example of the rich interplay between epitaxial strain and oxygen vacancies, which in turn may enormously influence the prevalent orbital and structural order parameters, is provided by the archetypal oxide perovskite $\hbox {SrTiO}_{{3}}$ (STO).

Bulk STO is a quantum paraelectric crystal with an almost ideal cubic perovskite structure and high dielectric constant^26^. Bulk STO is broadly used as a substrate in which to grow epitaxial perovskite thin films of high quality and as a key component of oxide heterostructures exhibiting fundamentally intriguing physical behaviour (e.g., $\hbox {LaTiO}_{{3}}$/STO bilayers, in which a 2D electron gas appears at the interface^27^, and $\hbox {PbTiO}_{{3}}$/STO superlattices, in which polar vortices have been observed^28^). At room temperature, STO epitaxial thin films may display in-plane ferroelectricity upon biaxial tensile stress^29^ and out-of-plane ferroelectricity upon biaxial compressive stress^30^. Within a certain range of biaxial compressive stress, antiferrodistortive (AFD) oxygen octahedra rotations are observed to coexist with out-of-plane electric polarization^30^, thus pointing to the presence of unusual cooperative couplings between such typically opposing order parameters^31^.

The interplay between oxygen vacancies and biaxial stress in STO thin films is very rich and appears to be further enhanced by the coexistence of AFD distortions and ferroelectricity^32,33^. Experimentally, it has been shown that both compressive and tensile biaxial strains significantly decrease the formation enthalpy of oxygen vacancies (e.g., by $\sim 10$% for $|\eta | \sim 1$%)^34^. This behaviour is different from zero-temperature first-principles results (also referred to as *ab initio*) obtained for other prototype oxide perovskites like $\hbox {BaTiO}_{{3}}$ and $\hbox {PbTiO}_{{3}}$, which indicate that compressive (tensile) biaxial stress typically depletes (promotes) the formation of ${\mathrm{V}_{{\mathrm{O}}}}$^35,36^. Meanwhile, atomic force microscopy experiments have shown that tensile biaxial strain produces a substantial increase in the mobility of oxygen vacancies whereas small compressive biaxial strain produces an incipient ${\mathrm{V}_{{\mathrm{O}}}}$ diffusion reduction^37^. These latter experimental observations appear to be in partial disagreement with previous zero-temperature first-principles studies in which it has been concluded that both tensile and compressive biaxial strains tend to promote the migration of oxygen ions^38,39^.

First-principles calculations have been used to rationalize the atomistic mechanisms of strain-mediated formation of oxygen vacancies for a number of oxide perovskite thin films like $\hbox {BaTiO}_{{3}}$^35^, $\hbox {PbTiO}_{{3}}$^36^, $\hbox {CaMnO}_{{3}}$^40^, and $\hbox {SrCoO}_{{3}}$^25,41^. As mentioned above, zero-temperature *ab initio* calculations, which by definition neglect thermal excitations, mostly agree in that the formation of ${\mathrm{V}_{{\mathrm{O}}}}$ is strongly enhanced (reduced) by tensile (compressive) epitaxial strain. This behaviour has been explained in terms of an effective decrease in the electrostatic repulsive interactions between electronically reduced TM ions, which follows from an increase in the average distance between them^40^. However, the nonmonotonic peak-like $\eta$-dependence of the ${\mathrm{V}_{{\mathrm{O}}}}$ formation enthalpy that has been measured for biaxially strained STO thin films^34^ cannot be satisfactorily explained in terms of such electrostatic arguments (as otherwise the formation energy of oxygen vacancies should increase, rather than decrease, under compressive $\eta$ conditions). Likewise, the partial disagreements between theory and experiments on the diffusion properties of oxygen vacancies^37–39^ appear to suggest that some key elements might be missing in previous *ab initio* studies^41^.

Here, we present a comprehensive first-principles study on the formation energy and migration of oxygen ions in epitaxially strained (001) STO thin films that explicitly incorporates lattice thermal effects. This improvement is achieved by means of quasi-harmonic free energy approaches and *ab initio* molecular dynamics (AIMD) simulations ("Computational methods" section). In particular, we compare the formation energies and energy barriers for oxygen diffusion estimated both at zero temperature and $T \ne 0$ conditions, and discuss their agreement with the available experimental data. It is found that thermal lattice excitations are necessary to qualitatively reproduce the measured dependence of ${\mathrm{V}_{{\mathrm{O}}}}$ formation energy on biaxial stress. Thermal lattice excitations are also found to enhance O ion migration by reducing the involved energy barriers in about 40%. In agreement with the experiments, the diffusion coefficient of oxygen ions is found to substantially increase under tensile biaxial stress and to decrease under compressive biaxial stress. The present works evidences the need to use finite-temperature first-principles methods to rationalize the experimental findings on off-stoichiometric oxide perovskite thin films and to guide the engineering of new functional materials based on combined physico-chemical approaches.

## Computational methods

### General technical details

We used the generalised gradient approximation to density functional theory (DFT) due to Perdew, Burke, and Ernzerhof (GGA-PBE)^42^ as is implemented in the VASP software^43^. A “Hubbard-*U*” scheme^44^ was employed for a better treatment of the Ti 3*d* electronic orbitals with a selected *U* value of 2.0 eV. (The main conclusions presented in this article do not appreciably depend on this particular choice as demonstrated by numerical tests carried out for $U = 4$ eV, see Supplementary Fig. 1 and "Formation energy of oxygen vacancies at T = 0" section.) The energy band gap of (001) $\hbox {SrTiO}_{{3}}$ thin films was accurately estimated with the range-separated hybrid functional HSE06^45^ for the equilibrium structures previously determined at the GGA-PBE+*U* level. We used the “projector augmented wave” method^46^ to represent the ionic cores and considered the following electronic states as valence: Sr 4*s*, 4*p* and 5*s*; Ti 3*p*, 4*s* and 3*d*; O 2*s* and 2*p*. Wave functions were represented in a plane-wave basis truncated at 650 eV.

For simulation of the stoichiometric systems, we employed a 20-atoms simulation cell that allows to reproduce the usual ferroelectric and $\hbox {O}_{{6}}$ antiferrodistortive (AFD) distortions in perovskite oxides^8,16^ (Fig. 1). Off-stoichiometric systems containing oxygen vacancies, ${\mathrm{V}_{{\mathrm{O}}}}$, were generated by removing oxygen atoms from either equatorial (Eq) or apical (Ap) positions (Fig. 1). Simulation cells of different sizes were considered in order to quantify the effects of oxygen vacancy concentration on the obtained formation energy results. In particular, the following compositions were investigated: $\hbox {Sr}_{{4}}\hbox {Ti}_{{4}}\hbox {O}_{{11}}$ (or, equivalently, $\hbox {SrTiO}_{2.75}$), $\hbox {Sr}_{{8}}\hbox {Ti}_{{8}}\hbox {O}_{{23}}$ ($\hbox {SrTiO}_{2.88}$), and $\hbox {Sr}_{{16}}\hbox {Ti}_{{16}}\hbox {O}_{{47}}$ ($\hbox {SrTiO}_{2.94}$). For integrations within the Brillouin zone (BZ), we used a $\Gamma$-centered $\mathbf{k}$-point grid of $6 \times 8 \times 8$ for the 20-atoms simulation cell and scaled it conveniently to maintain an equivalent $\mathbf{k}$-point density for the rest of cases. All oxygen vacancies were assumed to be neutrally charged and non-magnetic (${\mathrm{V}_{{\mathrm{O}}}}$) as in previous DFT studies this configuration has been shown to render the lowest energy for bulk off-stoichiometric $\hbox {SrTiO}_{{3}}$^9,47,48^.

The geometry relaxations of epitaxially strained (001) $\hbox {SrTiO}_{{3}}$ and $\hbox {SrTiO}_{3-\delta }$ were carried out by using a conjugated gradient algorithm that allowed to change the simulation-cell volume and atomic positions while constraining the length and orientation of the two in-plane lattice vectors (that is, $|a|=|b|$ and $\gamma = 90^{\circ }$). Periodic boundary conditions were applied along the three lattice-vector directions, thus the influence of surface effects were systematically neglected in our simulations. This type of calculations are known as “strained-bulk” geometry relaxations and typically are considered to be a good approximation for thin films presenting thicknesses of at least few nanometers^8,15–17,19^. The simulated systems were assumed to be elastically coupled to a substrate thus the existence of possible stress relaxation mechanisms in the thin films were also neglected. The geometry relaxations were stopped when the forces on the ions were smaller than 0.01 eV/Å. By using these parameters we obtained zero-temperature energies that were converged to within 0.5 meV per formula unit.

The electric polarization of stoichiometric and off-stoichiometric (001) $\hbox {SrTiO}_{{3}}$ thin films were estimated with the Born effective charges method^8,16^. In this approach, the electric polarization is calculated via the formula:

1 $$\begin{aligned} P_{\alpha } = \frac{1}{\Omega } \sum _{\kappa \beta } Z_{\kappa \beta \alpha }^{*} u_{\kappa \beta }, \end{aligned}$$

where $\Omega$ is the volume of the cell, $\kappa$ runs over all the atoms, $\alpha ,\beta = x, y, z$ represent the Cartesian directions, ${\mathbf {u}}_{\kappa }$ is the displacement vector of the $\kappa$-th atom as referred to a non-polar reference phase, and ${{Z}}^{*}_{\kappa }$ the Born effective charge tensor calculated for a non-polar reference state. It is worth noting that the presence of oxygen vacancies typically induced a notable reduction in the energy band gap of off-stoichiometric systems, which in some cases led to the appearence of metallic states. Consequently, estimation of the electric polarization with the more accomplished and accurate Berry phase formalism was not possible for all the analyzed compositions and thus we opted for systematically using the approximate Born effective charges method^8,49^.

### Phonon calculations

To estimate phonon frequencies we employed the “small-displacement” approach^50^, in which the force-constant matrix of the crystal is calculated in real space by considering the proportionality between the atomic displacements and forces when the former are sufficiently small (in the present study this condition was satisfied for atomic displacements of 0.02 Å). Large supercells containing 160 atoms were employed to guarantee that the elements of the force-constant matrix presented practically negligible values at the largest atomic separations. We used a dense $\mathbf{k}$-point grid of $3 \times 3 \times 3$ for the calculation of the atomic forces with VASP. The computation of the nonlocal parts of the pseudopotential contributions were performed in reciprocal space in order to maximise the numerical accuracy. Once a force-constant matrix was determined, we Fourier transformed it to obtain the phonon frequencies for any arbitrary $\mathbf{k}$-point in the first BZ. This latter step was performed with the PHON code^50^, in which the translational invariance of the system is exploited to ensure that the three acoustic branches are exactly zero at the $\Gamma$ point. Central differences for the atomic forces, that is, both positive and negative atomic displacements, were considered. A complete phonon calculation involved the evaluation of atomic forces for 120 (114) different stoichiometric (off-stoichiometric) configurations with the technical parameters just described. In order to accurately compute $F^{{{\mathrm{qh}}}}_{\mathrm{vac}}$ (see below), we employed a dense $\mathbf{k}$-point grid of $16 \times 16 \times 16$ for BZ integration. With these settings we found that the calculated quasi-harmonic free energies were accurate to within 5 meV per formula unit. Examples of full phonon spectra calculated for epitaxially strained stoichiometric and non-stoichiometric STO are provided in the Supplementary Fig. 2.

### Free energy calculations

We computed the quasi-harmonic Gibbs free energy associated with the formation of neutral oxygen vacancies, $G^{{{\mathrm{qh}}}}_{\mathrm{vac}}$, as a function of epitaxial strain, $\eta \equiv \left( a - a_{0}\right) / a_{0}$ (where $a_{0}$ represents the equilibrium in-plane lattice parameter calculated for the stoichiometric system), and temperature, *T*, by following the approach introduced in previous works^25,41^. Next, we briefly summarize the key aspects and technical details of the employed quasi-harmonic Gibbs free energy calculation method.

The formation Gibbs free energy of non-magnetic and neutrally charged ${\mathrm{V}_{{\mathrm{O}}}}$ can be expressed as^25,41^:

2 $$\begin{aligned} G^{{{\mathrm{qh}}}}_{\mathrm{vac}}(\eta , T) = E_{\mathrm{vac}}(\eta ) + F^{{{\mathrm{qh}}}}_{\mathrm{vac}}(\eta , T) + \mu _{\mathrm{O}}(T), \end{aligned}$$

where subscript “vac” indicates the quantity difference between the off-stoichiometric and stoichiometric systems (e.g., $E_{\mathrm{vac}} \equiv E_{{\mathrm{SrTiO}}_{3-\delta }} - E_{\mathrm{SrTiO}_{3}}$), $E_{\mathrm{vac}}$ accounts for the static contributions to the free energy (i.e., calculated at $T = 0$ conditions by considering the atoms fixed at their equilibrium lattice positions^8^), $F^{{{\mathrm{qh}}}}_{\mathrm{vac}}$ for the vibrational contributions to the free energy, and $\mu _{\mathrm{O}}$ is the chemical potential of free oxygen atoms. The vibrational free energy of stoichiometric and off-stoichiometric systems were estimated with the quasi-harmonic formula^51–55^:

3 $$\begin{aligned} F^{{{\mathrm{qh}}}}(\eta , T) = \frac{1}{N_{q}}~k_{\mathrm{B}} T \sum _{\mathbf{q}s}\ln \left[ 2\sinh \left( \frac{\hbar \omega _{\mathbf{q}s}(\eta )}{2k_{\mathrm{B}}T} \right) \right] , \end{aligned}$$

where $N_{q}$ is the total number of wave vectors used for integration within the Brillouin zone and the dependence of the phonon frequencies, $\omega _{{{q}}s}$, on epitaxial strain is explicitly noted.

It is well known that first-principles estimation of $\mu _{\mathrm{O}}$ with DFT$+U$ methods is challenging and may lead to large errors^56,57^. Such inherent limitations make the prediction of ${\mathrm{V}_{{\mathrm{O}}}}$ formation energies by exclusively using DFT approaches difficult and probably also imprecise. Notwithstanding, since (i) the chemical potential of free oxygen atoms does not depend on the epitaxial strain conditions but on the experimental thin film synthesis conditions, and (ii) our main goal here is to unravel the impact of lattice excitations on the formation energy and diffusion of ${\mathrm{V}_{{\mathrm{O}}}}$ as a function of $\eta$, we can safely base our analysis on the results obtained for the thermodynamically shifted Gibbs free energy:

4 $$\begin{aligned} G^{* {{{\mathrm{qh}}}}}_{\mathrm{vac}}(\eta , T) = G^{{{\mathrm{qh}}}}_{\mathrm{vac}}(\eta , T) - \mu _{\mathrm{O}}(T)~. \end{aligned}$$

In other words, rather than adopting experimental values for $\mu _{\mathrm{O}}$ and/or applying empirical corrections to the calculated vacancy formation energies^40,57^, here we select arbitrary values for the oxygen-gas chemical potential without any loss of generality.

### Nudged elastic band calculations

*Ab initio* nudged-elastic band (NEB) calculations^58^ were performed to estimate the activation energy for ${\mathrm{V}_{{\mathrm{O}}}}$ diffusion in expitaxially strained (001) $\hbox {SrTiO}_{{3}}$ at zero temperature. Our NEB calculations were performed for reasonably large $2 \times 2 \times 2$ or $3 \times 3 \times 3$ supercells containing several tens of atoms^47^. We used $\mathbf{q}$-point grids of $8 \times 8 \times 8$ or $6 \times 6 \times 6$ and an energy plane-wave cut-off of 650 eV. Six intermediate images were used to determine the energy barrier for oxygen diffusion along the most likely ${\mathrm{V}_{{\mathrm{O}}}}$ diffusion paths in the absence of thermal excitations. The geometry optimizations were halted when the total forces on the atoms were smaller than 0.01 eV$\cdot$Å$^{-1}$. The NEB calculations were performed for five epitaxial-strain equidistant points in the interval $-4 \le \eta \le 4$%.

### *Ab initio* molecular dynamics simulations

First-principles molecular dynamics (AIMD) simulations based on DFT were performed in the canonical (*N*, *V*, *T*) ensemble. The selected volumes and geometries were those determined at zero-temperature conditions, hence we neglected thermal expansion effects. The concentration of oxygen vacancies in the off-stoichiometric systems was also considered to be independent of *T* and equal to $\approx 1.6$%. The temperature in the AIMD simulations was kept fluctuating around a set-point value by using Nose-Hoover thermostats. Large simulation boxes containing 317 atoms ($\hbox {Sr}_{{64}}\hbox {Ti}_{{64}}\hbox {O}_{{189}}$) were employed in all the AIMD simulations and periodic boundary conditions were applied along the three Cartesian directions. Newton’s equation of motion were integrated by using the customary Verlet’s algorithm and a time-step length of $\delta t = 10^{-3}$ ps. $\Gamma$-point sampling for integration within the first Brillouin zone was employed in all the AIMD simulations. The calculations comprised total simulation times of $t_{total} \sim 10$ ps. We performed three AIMD simulations at $T = 1000$, 1500, and 2000 K for off-stoichiometric STO thin films considering epitaxial strains of $-3.6$, 0 and $+3.6$%.

The mean square displacement (MSD) of oxygen ions was estimated with the formula^59^:

5 $$\begin{aligned} {\mathrm{MSD}}(\tau )= & {} \frac{1}{N_{ion} \left( N_{step} - n_{\tau } \right) } \nonumber \\&\times \sum _{i=1}^{N_{ion}} \sum _{j=1}^{N_{step} - n_{\tau }} | {{\mathbf{r}}}_{i} (t_{j} + \tau ) - {{\mathbf{r}}}_{i} (t_{j}) |^{2}, \end{aligned}$$

where ${\mathbf{r}}_{i}(t_{j})$ is the position of a migrating ion *i* at time $t_{j}$ ($= j \cdot \delta t$), $\tau$ represents a lag time, $n_{\tau } = \tau / \delta t$, $N_{ion}$ is the total number of mobile ions, and $N_{step}$ the total number of time steps. The maximum $n_{\tau }$ was chosen equal to $N_{step}/3$ (i.e., equivalent to $\sim 3$–4 ps), hence we could accumulate enough statistics to reduce significantly the ${\mathrm{MSD}}(\tau )$ fluctuations at the largest $\tau$ (see the error bars in the ${\mathrm{MSD}}$ plots presented in the following sections). Oxygen diffusion coefficients were subsequently obtained with the Einstein relation:

6 $$\begin{aligned} D = \lim _{\tau \rightarrow \infty } \frac{{\mathrm{MSD}}(\tau )}{6\tau }~. \end{aligned}$$

The *T*-dependence of the oxygen diffusion coefficient was assumed to follow the Arrhenius formula:

7 $$\begin{aligned} D(T) = D_{0} \cdot \exp {\left[ -\frac{E_{a}}{k_{B}T} \right] }, \end{aligned}$$

where $D_{0}$ is known as the pre-exponential factor, $E_{a}$ is the activation energy for ionic diffusion, and $k_{B}$ the Boltzmann constant.

**Figure 2:**
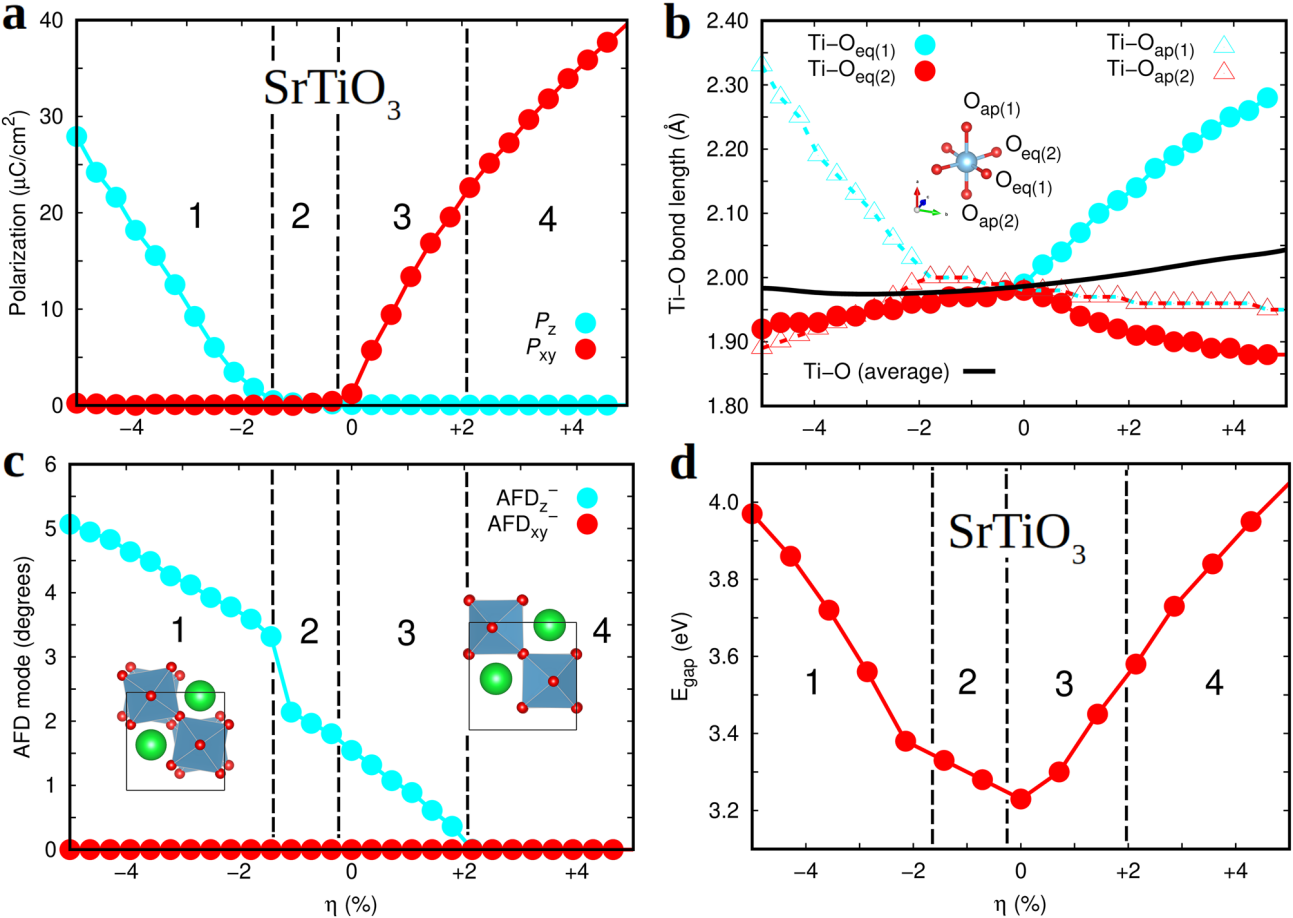
Physical properties of stoichiometric (001) $\hbox {SrTiO}_{{3}}$ thin films estimated with first-principles methods. (**a**) The electric polarization along the out-of-plane ($P_{z}$) and in-plane ($P_{xy}$) directions expressed as a function of expitaxial strain, $\eta$. (**b**) Different Ti–O bond lengths expressed as a function of epitaxial strain. (**c**) Antiphase out-of-plane ($\hbox {AFD}_{z}^{-}$) and in-plane ($\hbox {AFD}_{xy}^{-}$) antiferrodistortive $\hbox {O}_{{6}}$ rotations expressed as a function of epitaxial strain. (**d**) Energy band gap of (001) $\hbox {SrTiO}_{{3}}$ thin films estimated with the range-separated hybrid functional HSE06^45^ on the geometries determined at the PBE+*U* level. The vertical lines indicate different phase stability regions, namely, 1. *I*4*cm*, 2. *I*4/*mcm*, 3. *Ima*2 and 4. *Amm*2. The solid lines are simply guides for the eye.

## Results and discussion

We start by discussing the zero-temperature phase diagram of stoichiometric (001) STO thin films calculated with first-principles methods. The changes in the structural and electric polarization properties induced by the presence of equatorial (Eq) and apical (Ap) oxygen vacancies (${\mathrm{V}_{{\mathrm{O}}}}$) are subsequently explained. The impact of thermal effects on the formation energy of ${\mathrm{V}_{{\mathrm{O}}}}$ is analyzed for a wide range of epitaxial strain ($-4 \lesssim \eta \lesssim +4$%) and temperature ($0 \le T \le 1000$ K) conditions. We also report and compare the energy barriers for ionic oxygen diffusion in (001) STO thin films estimated by neglecting and by taking into account *T*-induced lattice vibrations. Insightful connections between our theoretical results and experimental measurements are provided whenever the latter are available.

It is worth noting that in experiments the epitaxial strains that are usually achieved are of the order of 1–2% since under larger $|\eta |$’s oxide perovskites tend to elastically relax via the formation of dislocations and other structural imperfections^60^. Nevertheless, misfit strain relaxation in oxide perovskites can be precluded, to some extent, through modulation of the thin film thickness and compositional engineering^61,62^. For instance, an experimentally attained tensile epitaxial strain of $+3.9$% has been recently reported for oxygen-deficient $\hbox {SrCoO}_{3-\delta }$ thin films^25^. The epitaxial strain conditions considered in the present computational study, therefore, aim to reproduce the broad $|\eta |$ ranges realized in experiments.

### Zero-temperature properties of stoichiometric (001) $\hbox {SrTiO}_{{3}}$ thin films

Figure 2 shows the structural, electric polarization and energy band gap properties of stoichiometric (001) $\hbox {SrTiO}_{{3}}$ thin films estimated with first-principles methods (i.e., density functional theory –DFT–, "Computational methods" section) at zero temperature. Different crystalline phases are stabilized as a result of varying the epitaxial strain conditions, which are described in detail next. We note that several authors have previously reported analogous DFT results to ours^15,63–65^ and that the best agreement with the present calculations is obtained for work^65^, in which antiferrodistortive oxygen octahedra rotations (AFD) were also explicitly modeled.

In the epitaxial strain interval $\eta \lesssim -2$%, we observe the stabilization of a tetragonal *I*4*cm* phase that is characterized by a significant out-of-plane polarization ($P_{z}$, Fig. 2a) and antiphase out-of-plane $\hbox {O}_{{6}}$ rotations ($\hbox {AFD}_{z}^{-}$, Fig. 2c). Coexistence of the order parameters $P_{z}$ and $\hbox {AFD}_{z}^{-}$ is quite unique as they normally tend to oppose each other^31^, a polar-antiferrodistortive interplay that has been experimentally observed and characterized as a function temperature and $\eta$^30^. Under large compressive strain, half of the Ti–O bond lengths involving oxygen atoms in apical positions are significantly elongated as compared to those involving O ions in equatorial positions (Fig. 2b), a structural distortion that signals the presence of out-of-plane polarization^16,18^. In the epitaxial strain interval $-2 \lesssim \eta \lesssim 0$%, a tetragonal *I*4/*mcm* phase appears that presents null electric polarization (Fig. 2a) and moderate antiphase out-of-plane $\hbox {O}_{{6}}$ rotations (Fig. 2c). In this phase, the length of the Ti–O bonds are all pretty similar regardless of the positions occupied by the oxygen atoms (Fig. 2b). It is worth noting that when some tiny monoclinic lattice distortions in the generated equilibrium geometries (i.e., $\alpha \sim 0.1$ degrees) are not disregarded the identification of this phase is also compatible with a non-polar *C*2/*c* phase that has been recently predicted for metallic $\hbox {LaNiO}_{{3}}$ thin films^66^.

In the epitaxial strain interval $0 \lesssim \eta \lesssim +2$%, a noticeable in-plane electric polarization, $P_{xy}$, appears in the system that coexists with small $\hbox {AFD}_{z}^{-}$ $\hbox {O}_{{6}}$ rotations (Fig. 2a,c). The resulting crystal phase is orthorhombic and its symmetry can be ascribed to the polar space group *Ima*2. Under tensile strain, half of the Ti–O bond lengths involving oxygen atoms in equatorial positions are significantly elongated as compared to those involving O ions in apical positions (Fig. 2b), a structural distortion that produces a significant in-plane polarization^16,18^. In the epitaxial strain interval $\eta \gtrsim +2$%, the antiphase out-of-plane $\hbox {O}_{{6}}$ rotations completely disappear and $P_{xy}$ grows steadily under increasing epitaxial strain. In this latter case, the optimized crystal structure is also orthorhombic and its symmetry can be identified with the space group *Amm*2.

Figure 2d shows the energy band gap of (001) $\hbox {SrTiO}_{{3}}$ thin films, $E_{\mathrm{gap}}$, estimated as a function of epitaxial strain with the range-separated hybrid functional HSE06^45^. The reason for including this information here will become clearer in the next subsection, where we explain the oxygen vacancy formation energy results obtained at zero temperature. It is worth noting that $E_{\mathrm{gap}}$ increases noticeably under either tensile or compressive biaxial strain as compared to the corresponding zero-strain value. For instance, at $\eta = 0$ the energy band gap amounts to 3.2 eV whereas at $\eta = \pm 4$% is approximately equal to 3.9 eV. Such a $\eta$-induced $E_{\mathrm{gap}}$ trend is markedly different from the one predicted for binary oxides like $\hbox {CeO}_{{2}}$ and $\hbox {TiO}_{{2}}$ by using analogous first-principles methods^21^, which displays a significant $E_{\mathrm{gap}}$ reduction under tensile biaxial strain. The reason for such a difference in $E_{\mathrm{gap}}$ behaviour is likely to be related to the larger changes in the dielectric susceptibility that can be induced by epitaxial strain in STO thin films as compared to binary oxides^21,63^.

**Figure 3:**
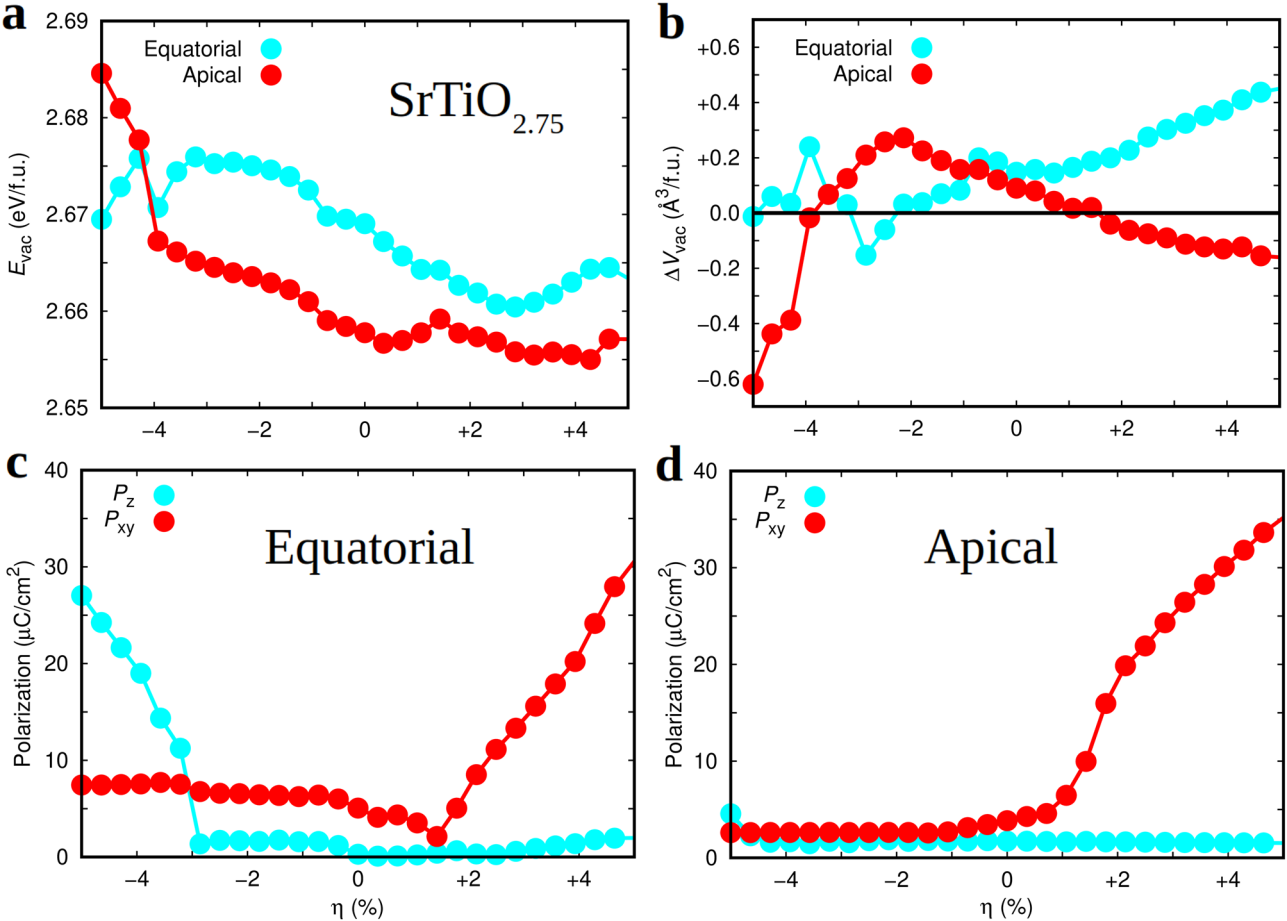
Zero-temperature properties of non-stoichiometric epitaxially strained (001) $\hbox {SrTiO}_{2.75}$ estimated with first-principles methods based on DFT ("Computational methods" section). (**a**) Zero-temperature formation energy of oxygen vacancies expressed as a function of oxygen position and epitaxial strain. (**b**) Volume change per formula unit, $\Delta V_{\mathrm{vac}} \equiv V_{{\mathrm{SrTiO}}_{2.75}} - V_{\mathrm{SrTiO}_{3}}$, induced by the creation of oxygen vacancies and expressed as a function of oxygen position and epitaxial strain. The electric polarization along the out-of-plane ($P_{z}$) and in-plane ($P_{xy}$) directions expressed as a function of expitaxial strain for (001) $\hbox {SrTiO}_{2.75}$ thin films containing (**c**) equatorial and (**d**) apical oxygen vacancies. The solid lines are simply guides for the eye.

### Formation energy of oxygen vacancies at $T = 0$

Figure 3 shows the formation energy of oxygen vacancies calculated for (001) STO thin films at zero temperature, $E_{\mathrm{vac}}$. The concentration of ${\mathrm{V}_{{\mathrm{O}}}}$ considered in this case renders the composition $\hbox {SrTiO}_{2.75}$ (analogous vacancy energy results obtained for smaller oxygen vacancy concentrations are explained below). A small decrease in $E_{\mathrm{vac}}$ is observed as the biaxial strain changes from compressive to tensile (i.e., of $> 1$% when considering the two limiting cases $\eta = \pm 4$%, Fig. 3a). For most $\eta$ cases, it seems more favourable to create apical ${\mathrm{V}_{{\mathrm{O}}}}$ than equatorial, however the formation energy differences between the two cases are pretty small (i.e., $|\Delta E_{\mathrm{vac}}| \approx 0.01$ eV per formula unit, Fig. 3a).

We repeated the $E_{\mathrm{vac}} \left( \eta \right)$ calculations shown in Fig. 3a by adopting a larger value of the technical parameter *U* (i.e., equal to 4 eV, "General technical details" section) and found quantitatively equivalent results to the ones just explained for $U = 2$ eV (Supplementary Fig. 1). In particular, the creation of apical oxygen vacancies was found to be energetically most favourable for most of the investigated $\eta$’s and the formation energy differences between apical and equatorial ${\mathrm{V}_{{\mathrm{O}}}}$’s were also of the order of 0.01 eV. Moreover, analogous $E_{\mathrm{vac}}$ results were obtained by (i) considering the presence of long-range dispersion interactions^67–70^ and (ii) employing other exchange-correlation DFT functionals like the popular local density approximation (LDA^71^) and the recently proposed meta-GGA functional SCAN^72,73^ (Supplementary Fig. 1). The study of the $\eta$-dependence of the formation energy of oxygen vacancies in STO thin films, therefore, appears to be quite insensitive to the particular choice of the *U* parameter and even also of the adopted DFT exchange-correlation functional. This outcome is likely to be a consequence of large error cancellations between the energies calculated for stoichiometric and non-stoichiometric systems^8^. Hereafter, all the reported results correspond to PBE+*U* ($U = 2$ eV) calculations.

For negative $\eta$ values, the creation of oxygen vacancies in either equatorial (Fig. 3c) or apical (Fig. 3d) positions has a dramatic effect on the electric polarization of the system. In particular, the sizable out-of-plane polarization found in stoichiometric STO thin films (Fig. 2a) practically disappears when ${\mathrm{V}_{{\mathrm{O}}}}$ are exclusively created in apical positions. Meanwhile, when oxygen vacancies are generated solely in equatorial positions a non-negligible in-plane polarization of $\approx 7$ $\mu$C cm$^{-2}$ appears for any value of compressive epitaxial strain. For positive $\eta$ values, on the other hand, the general behaviour of the electrical polarization is quite similar to that found for the analogous stoichiometric thin films, although the size of $P_{xy}$ appreciably decreases ($\sim 10$%).

Figure 3b shows the volume difference between $\hbox {SrTiO}_{2.75}$ and stoichiometric thin films, $\Delta V_{\mathrm{vac}}$, expressed as a function of epitaxial strain. The creation of neutral oxygen vacancies in oxide perovskites typically induces an increase in volume, the so-called chemical expansion, due to the electronic reduction of transition metal ions that are located close to ${\mathrm{V}_{{\mathrm{O}}}}$’s^41,74^. For present purposes, it is interesting to analyze the $\eta$–dependence of $\Delta V_{\mathrm{vac}}$ because this quantity has been found to be correlated with the contribution of lattice thermal excitations to the formation energy of ${\mathrm{V}_{{\mathrm{O}}}}$ at finite temperatures^41^. As regards equatorial oxygen vacancies, $\Delta V_{\mathrm{vac}}$ turns out to be positive and moderately large (small) under tensile (compressive) epitaxial strain. By contrast, the creation of apical oxygen vacancies is accompanied by negative (positive) and large $\Delta V_{\mathrm{vac}}$ absolute values (small) at large compressive (tensile) epitaxial strain (Fig. 3b). In the next subsection, we will comment on possible correlations between these zero-temperature $\Delta V_{\mathrm{vac}}$ results and the lattice-related contributions to the formation energy of ${\mathrm{V}_{{\mathrm{O}}}}$ at finite temperatures (i.e., the Gibbs free energy $G^{* {\mathrm{qh}}}_{\mathrm{vac}}$ shown in Eq.(4)).

**Figure 4:**
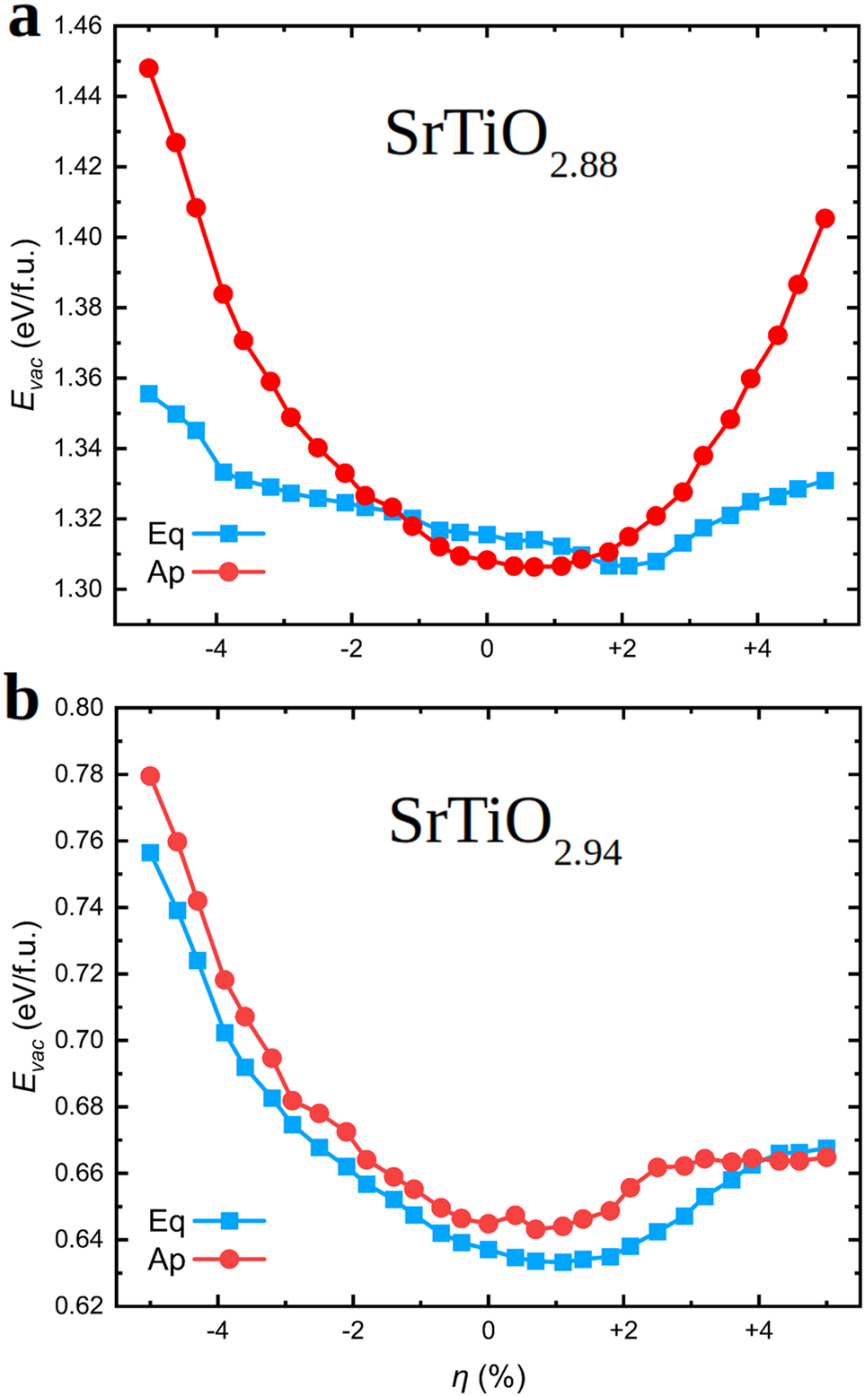
Zero-temperature formation energy of oxygen vacancies expressed as a function of oxygen position and epitaxial strain for compositions (**a**) $\hbox {SrTiO}_{2.88}$ and (**b**) $\hbox {SrTiO}_{2.94}$. Labels “Eq” and “Ap” stand out for equatorial and apical ${\mathrm{V}_{{\mathrm{O}}}}$, respectively. The solid lines are simply guides for the eye.

The estimation of oxygen vacancy formation energies may depend strongly on the concentration of ${\mathrm{V}_{{\mathrm{O}}}}$ considered in the simulations due to the presence of short- and long-ranged interactions acting between the defects^2,8^. Figures 3a and 4 explicitly show this effect, as it is found that by decreasing the ${\mathrm{V}_{{\mathrm{O}}}}$ concentration the computed zero-temperature formation energy dramatically decreases for any arbitrary value of $\eta$. For instance, the estimated $E_{\mathrm{vac}}$ for unstrained $\hbox {SrTiO}_{2.75}$ and $\hbox {SrTiO}_{2.94}$ amounts to 2.7 and 0.6 eV, respectively. This result suggests that short and middle-range interactions between oxygen vacancies are of repulsive type and thus the formation of ${\mathrm{V}_{{\mathrm{O}}}}$ clusters in STO thin films in principle is not likely to occur at low and moderate temperatures. Moreover, the $E_{\mathrm{vac}}$ difference between equatorial and apical oxygen vacancies also depends critically on the concentration of defects. Specifically, according to our $E_{\mathrm{vac}}$ results obtained for $\hbox {SrTiO}_{2.75}$ thin films in general it is more favourable to create apical ${\mathrm{V}_{{\mathrm{O}}}}$ than equatorial (Fig. 3a) whereas for $\hbox {SrTiO}_{2.94}$ thin films the tendency is just the opposite (Fig. 4b). The effect of epitaxial strain on $E_{\mathrm{vac}}$ also varies as the concentration of ${\mathrm{V}_{{\mathrm{O}}}}$ changes. In particular, $E_{\mathrm{vac}}$ increases both under compressive and tensile strains for $\hbox {SrTiO}_{2.94}$ thin films whereas for $\hbox {SrTiO}_{2.75}$ it decreases under tensile strain.

How do these zero-temperature ${\mathrm{V}_{{\mathrm{O}}}}$ formation energy results compare with the available experimental data? In a recent paper, Rivadulla and collaborators have measured the enthalpy of oxygen vacancy formation for STO thin films as a function of epitaxial stress^34^. The authors have found that under both compressive and tensile strains the ${\mathrm{V}_{{\mathrm{O}}}}$ formation energy noticeably decreases. For instance, in the experiments the ${\mathrm{V}_{{\mathrm{O}}}}$ formation enthalpy decreases by $\approx 20$% ($\approx 40$%) for a tensile (compressive) strain of 1% as compared to the unstrained case^34^. Therefore, the agreement between our zero-temperature $E_{\mathrm{vac}} (\eta )$ results and the experimental observations is far from satisfactory. We note that this conclusion is independent of the ${\mathrm{V}_{{\mathrm{O}}}}$ concentration considered in our simulations, as shown by Figs. 3a and 4. In order to fundamentally understand the origins of such large discrepancies, and based on the fact that oxygen vacancies in oxide perovskites typically are created at high temperatures^7,25,34^, we proceeded to explicitly calculate ${\mathrm{V}_{{\mathrm{O}}}}$ formation free energies at finite temperatures (rather than for non-realistic $T = 0$ conditions).

Before explaining our ${\mathrm{V}_{{\mathrm{O}}}}$ formation energy results obtained at $T \ne 0$ conditions, it is worth mentioning that in a recent work^33^ another first-principles study on the ${\mathrm{V}_{{\mathrm{O}}}}$ formation energy of STO thin films has been reported. Zero-temperature $E_{\mathrm{vac}}$ results analogous to ours are presented in^33^, however, the conclusions reported in that study are drastically different from the computational outcomes just described in this section. In particular, a systematic decrease in $E_{\mathrm{vac}}$ has been predicted for either tensile or compressive strains, which is the opposite behaviour than what we have found here for $\hbox {SrTiO}_{2.94}$ thin films, for instance. Moreover, an intriguing correlation between the $\eta$-induced behaviour of $E_{\mathrm{vac}}$ and the energy band gap of STO thin films ($E_{\mathrm{gap}}$) has been also suggested in work^33^. Based on our results enclosed in Figs. 2d and 4b, such a correlation is partially corroborated^21^. Nevertheless, in our calculations both quantities $E_{\mathrm{vac}}$ and $E_{\mathrm{gap}}$ increase, rather than decrease, under either tensile or compressive strains. We hypothesize that the likely reasons for such theoretical disagreements may be the neglection of characteristic STO structural motifs like polar and antiferrodistortive oxygen octahedral distortions in work^33^.

**Figure 5:**
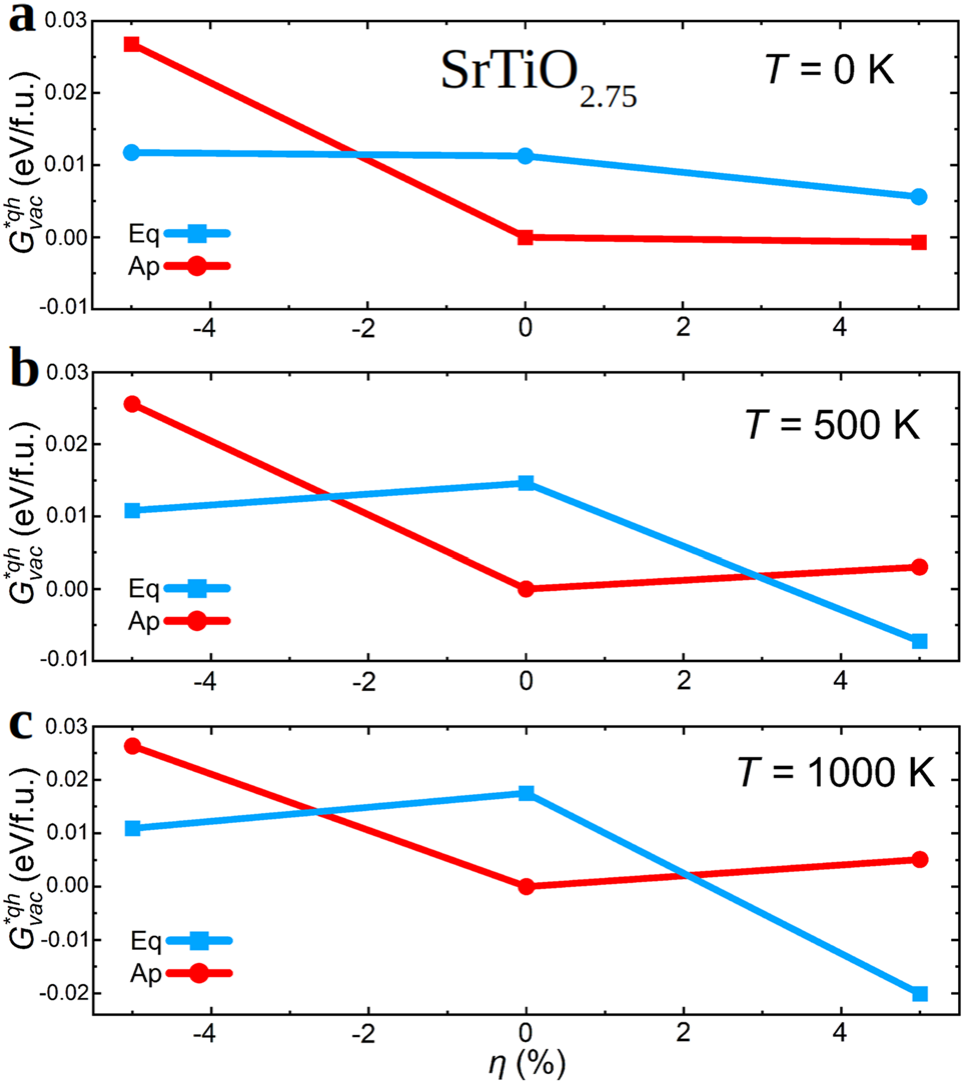
Thermodynamically $\mu _{\mathrm{O}}$-shifted Gibbs free energy [Eqs. (2)–(4)] (“Free energy calculations” section) for ${\mathrm{V}_{{\mathrm{O}}}}$ formation expressed as a function of oxygen vacancy position, epitaxial strain and temperature. (**a**) $T = 0$ K, (**b**) $T = 500$ K, and (**c**) $T = 1000$ K. The composition of the off-stoichiometric (001) thin films corresponds to $\hbox {SrTiO}_{2.75}$ and labels “Eq” and “Ap” stand out for equatorial and apical oxygen vacancies, respectively. The solid lines are simply guides for the eye.

### Formation energy of oxygen vacancies at $T \ne 0$

We performed quasi-harmonic Gibbs free energy calculations to estimate the formation energy of oxygen vacancies at finite temperatures, $G^{* {\mathrm{qh}}}_{\mathrm{vac}}$ [Eq.(4)], for epitaxially constrained (001) $\hbox {SrTiO}_{2.75}$ thin films using the methods explained in "Free energy calculations" section. Due to obvious computational limitations associated with the calculation of the full phonon spectrum of defective crystals, the dependence of $G^{* {\mathrm{qh}}}_{\mathrm{vac}}$ on vacancy concentration could not be assessed. Figure 5 shows our $G^{* {\mathrm{qh}}}_{\mathrm{vac}}$ results expressed as as function of temperature and epitaxial strain. Since here we are primarily interested in analyzing the joint effects of epitaxial strain and lattice thermal excitations on the formation energy of oxygen vacancies, the chemical potential entering Eq.(4) has been arbitrarily selected, without any loss of generality, to provide null $G^{* {\mathrm{qh}}}_{\mathrm{vac}}$ values for the energy minimum determined at $\eta = 0$ and each temperature ("Free energy calculations" section).

We found that the $\eta$-dependence of the $\mu _{\mathrm{O}}$-shifted formation energy of equatorial ${\mathrm{V}_{{\mathrm{O}}}}$’s drastically changes as a result of considering *T*-induced lattice vibrations. For instance, at the highest analyzed temperature, $T =1000$ K, $G^{* {\mathrm{qh}}}_{\mathrm{vac}}$(Eq) decreases by $\approx 30$% for a biaxial strain of $-5$% and by $\approx 200$% for $\eta = +5$% as compared to the $T = 0$ unstrained case (Fig. 5c). For an intermediate temperature of 500 K, the observed tendency is analogous to the one just described although the $G^{* {\mathrm{qh}}}_{\mathrm{vac}}$(Eq) differences with respect to the $T = 0$ unstrained case are slightly smaller (i.e., a reduction of $\approx 15$% and $\approx 150$% for $\eta = -5$ and $+5$%, respectively—Fig.5b). Meanwhile, for apical oxygen vacancies $G^{* {\mathrm{qh}}}_{\mathrm{vac}}$(Ap) only changes moderately under tensile biaxial strains.

The differences between the estimated $G^{* {\mathrm{qh}}}_{\mathrm{vac}}$ as a function of *T* and $\eta$ for apical and equatorial ${\mathrm{V}_{{\mathrm{O}}}}$ can be qualitatively understood in terms of the zero-temperature proxy $\Delta V_{\mathrm{vac}}$ introduced in "Formation energy of oxygen vacancies at T = 0" section (Fig. 3b). In a recent theoretical paper^41^, it has been proposed that for positive $\Delta V_{\mathrm{vac}}$ values, that is, $V_{{\mathrm{SrTiO}}_{3-\delta }} > V_{\mathrm{SrTiO}_{3}}$, lattice thermal excitations tend to facilitate the formation of oxygen vacancies. As it is observed in Fig. 3b, for equatorial vacancies $\Delta V_{\mathrm{vac}}$ is positive and steadily increases under tensile biaxial strain; this outcome is agreeing with the large relative $G^{* {\mathrm{qh}}}_{\mathrm{vac}}$(Eq) decrease estimated for $\eta = +5$% upon increasing temperature (Fig. 5). Meanwhile, for apical vacancies $\Delta V_{\mathrm{vac}}$ is negative and may be small in absolute value under large tensile and compressive strains; this behaviour is consistent with the fact that under increasing temperature the corresponding relative $G^{* {\mathrm{qh}}}_{\mathrm{vac}}$(Ap) differences with respect to the $T = 0$ unstrained case only change slightly. Therefore, we corroborate the previously proposed qualitative correlation between the two quantities $\Delta V_{\mathrm{vac}}$ and $F^{{{\mathrm{qh}}}}_{\mathrm{vac}}$ ("Free energy calculations" section), which are computed at zero temperature and $T \ne 0$ conditions, respectively^41^.

How do these finite-temperature ${\mathrm{V}_{{\mathrm{O}}}}$ formation energy results compare with the experimental data reported in work^34^? The answer is that although the agreement between theory and observations is not quantitative it can be regarded as qualitatively satisfactory. We recall that experimentally it has been determined that under both compressive and tensile biaxial strains oxygen vacancies can be created more easily. This behaviour is analogous to what we have predicted for equatorial ${\mathrm{V}_{{\mathrm{O}}}}$’s, which in oxide perovskites correspond to the most representative class of anion positions (i.e., equatorial O sites are 50% more numerous than apical). Moreover, since the $G^{* {\mathrm{qh}}}_{\mathrm{vac}}$ values estimated for equatorial ${\mathrm{V}_{{\mathrm{O}}}}$’s under both tensile and compressive biaxial strains are smaller than those estimated for apical vacancies (by $\approx 30$ and 20 meV, respectively), it is likely that to a certain extent vacancy ordering occurs in epitaxially strained STO thin films (as it has been experimentally shown for grain boundaries in bulk STO from scanning transmission electron microscopy measurements^75^). On the down side, experiments indicate that it is more easy to create oxygen vacancies under compressive strain than under tensile strain^34^ while our calculations predict the opposite trend (Fig. 5). Nonetheless, based on our computational $E_{\mathrm{vac}}$ and $G^{* {\mathrm{qh}}}_{\mathrm{vac}}$ results, it can be concluded that in order to reproduce the experimentally observed $\eta$-induced enhancement of ${\mathrm{V}_{{\mathrm{O}}}}$ formation with theoretical *ab initio* methods it is necessary to explicitly consider vibrational lattice thermal excitations in the calculations.

**Figure 6:**
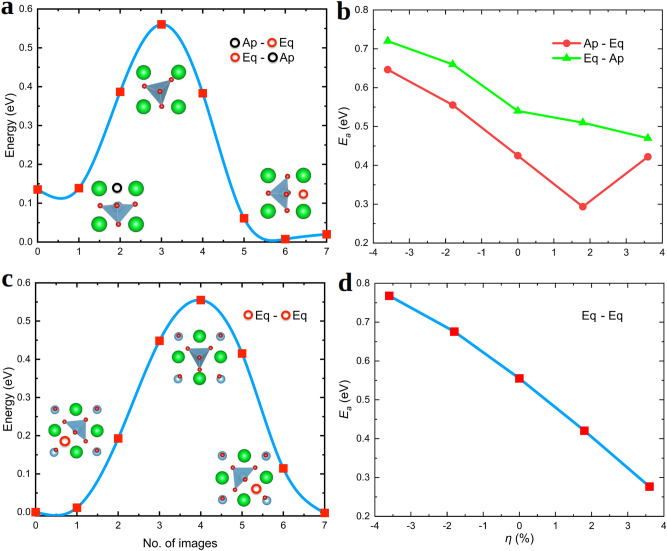
Energy barriers for ${\mathrm{V}_{{\mathrm{O}}}}$ diffusion calculated with the NEB method ("Nudged elastic band calculations" section) and by neglecting thermal lattice fluctuations. Representation of the analyzed oxygen vacancy diffusion paths are shown in (**a**) and (**c**) ($\eta = 0$ case). NEB energy barrier results expressed as a function of epitaxial strain are represented in (**b**) and (**d**). Labels “Eq” and “Ap” stand out for equatorial and apical oxygen vacancies, respectively. The colouring code for atoms in (**a**) and (**c**) coincides with that indicated in Fig. 1. The solid lines are simply guides for the eye.

**Figure 7:**
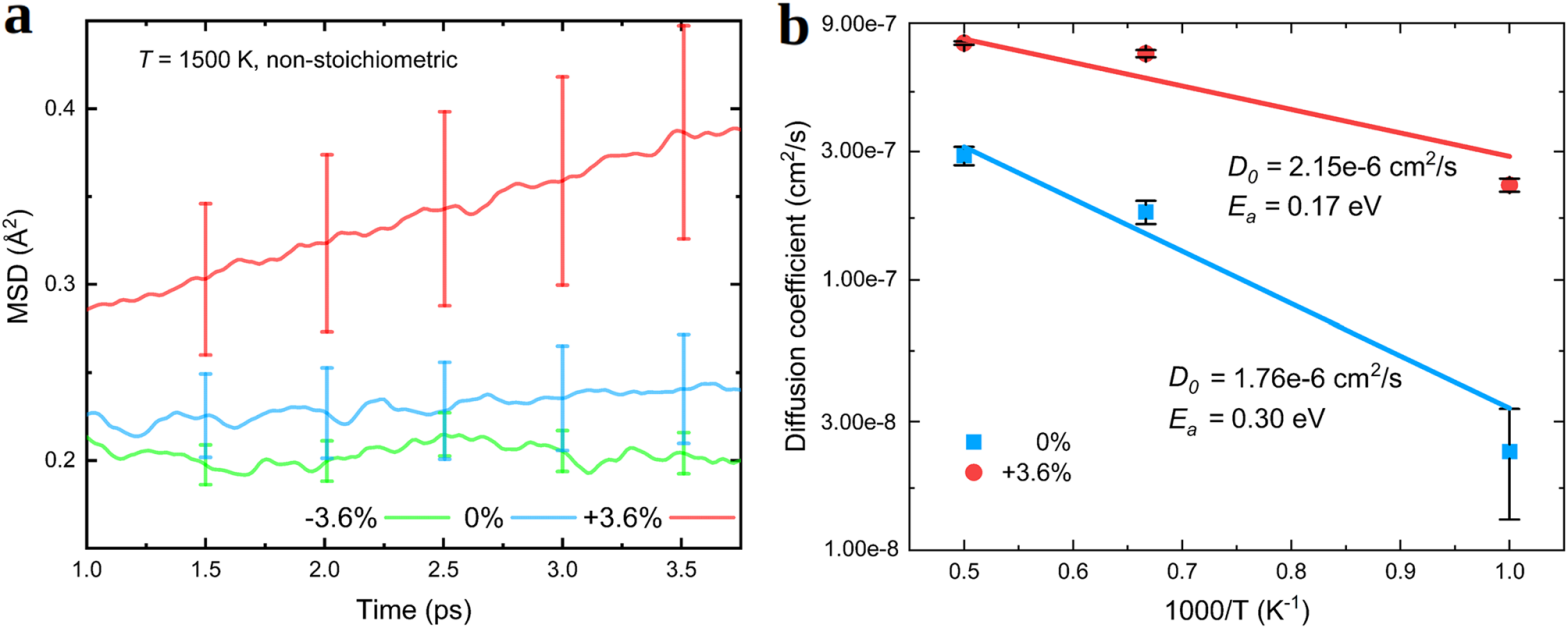
(**a**) Mean square displacement (MSD) calculated for oxygen ions in off-stoichiometric (001) STO thin films with AIMD simulations performed at $T = 1500$ K and considering different epitaxial strain conditions, namely, $\eta = -3.6$, 0 and $+3.6$%. (**b**) Oxygen diffusion coefficients estimated for off-stoichiometric (001) STO thin films with AIMD simulations considering different temperatures and epitaxial strain conditions. The resulting pre-exponential factors, $D_{0}$, and activation energies, $E_{a}$, for oxygen ionic diffusion are indicated in the plot ("Ab initio molecular dynamics simulations" section).

### Zero-temperature activation energy for oxygen diffusion

The diffusion of O ions in oxide perovskites is a key parameter for the design of ionic-based devices^76^. In recent atomic force microscopy experiments performed by Iglesias *et al.*^37^, it has been shown that tensile biaxial strain produces a substantial increase in the diffusion of O ions in STO thin films. In particular, the room-temperature diffusion coefficient of oxygen atoms, $D_{\mathrm{O}}$, roughly increases by a factor of 4 upon a tensile biaxial strain of $\approx +2$%^37^. For compressive tensile strains, on the other hand, the available experimental data is quite scarce. Nonetheless, measurements performed up to a $\eta$ of $\approx -1$% appear to suggest an incipient reduction in $D_{\mathrm{O}}$^37^. On this regard, first-principles analysis of ionic transport properties may be very useful as calculations are free of the technical issues encountered in the experimental synthesis of epitaxially grown thin films and thus arbitrarily large tensile/compressive biaxial strains can be simulated.

First-principles simulation of ionic diffusion processes, however, are neither exempt of some technical issues and shortcomings^59^. For instance, due to the intense computational expense associated with $T \ne 0$ simulations, most first-principles studies usually neglect temperature effects. In particular, zero-temperature calculations of ion-migration energy barriers typically are performed with the nudged elastic band (NEB) method^58^ ("Zero-temperature activation energy for oxygen diffusion" section), in which (i) the initial and final diffusion positions of the vacancy and interstitial ions need to be guessed in the form of high-symmetry configurations rendering metastable states, and (ii) *T*-induced lattice excitations are totally neglected. Limitations of the NEB method for accurately determining ionic diffusion energy barriers and paths are well known and documented for some prototype fast-ion conductor materials (e.g., see works^59^ and^77^).

Al-Hamadany *et al.* have already studied the migration of oxygen vacancies in (001) STO thin films by means of NEB and DFT methods^38,39^. For the case of tensile biaxial strains, Al-Hamadany *et al.* have reported a systematic and significant reduction in the energy barrier for ${\mathrm{V}_{{\mathrm{O}}}}$ diffusion, $E_{a}$ (i.e., of up to 25% for large $\eta$’s of $+6$–8%^39^). This computational outcome is in good agreement with the experimental tendency found by Iglesias *et al.* for $D_{\mathrm{O}}$^37^. The value of the reported NEB activation energy calculated at zero-strain conditions is approximately 0.8 eV. For the case of compressive biaxial strains, Al-Hamadany *et al.* have also reported a decrease in $E_{a}$ for high $|\eta |$’s of $> 4$%^38^ (i.e., of up to 50% for $\eta$’s of $-6$–8%); in the $0 \le \eta \le 4$ interval, on the other hand, the energy barrier for ${\mathrm{V}_{{\mathrm{O}}}}$ diffusion hardly changes or increases just moderately (depending on the considered initial and final oxygen vacancy positions).

Figure 6 shows our $E_{a}$ results obtained for (001) STO thin films by employing DFT NEB techniques ("Zero-temperature activation energy for oxygen diffusion" section). Two possible ${\mathrm{V}_{{\mathrm{O}}}}$ diffusion paths, namely, “Ap-Eq” (Fig. 6a) and “Eq–Eq” (Fig. 6c) where “Ap” and “Eq” stand for apical and equatorial O sites, have been considered in our simulations. In the former case, we obtain two different energy barriers, “Ap-Eq” and “Eq-Ap”, due to the energy asymmetry between the two involved oxygen positions (Figs. 3 and 4). In consistent agreement with the available experimental data and previous DFT studies, we find that under tensile biaxial strain the energy barrier for ${\mathrm{V}_{{\mathrm{O}}}}$ diffusion is greatly reduced. For instance, at $\eta \approx +4$% we obtain that $E_{a}$ decreases with respect to the value estimated at zero strain (i.e., 0.55 eV) by $\approx 50$% and 15% for “Eq–Eq” (Fig. 6d) and “Eq-Ap” (Fig. 6b), respectively. (The ${\mathrm{V}_{{\mathrm{O}}}}$ diffusion energy barrier difference between cases “Eq-Ap” and “Ap-Eq” simply correspond to the zero-temperature ${\mathrm{V}_{{\mathrm{O}}}}$ formation energy difference between cases “Eq” and “Ap”.) It is worth noting that our estimated zero-strain $E_{a}$ value of 0.55 eV is in very good agreement with the experimental ${\mathrm{V}_{{\mathrm{O}}}}$ diffusion energy barrier measured for bulk STO, $E_{a}^{\mathrm{expt}} \approx 0.60$ eV^78^.

Upon compressive biaxial strain, we find that $E_{a}$ increases significantly and practically linearly with $|\eta |$ (Fig. 6b,d). For instance, at $\eta \approx -4$% we predict that $E_{a}$ increases with respect to the zero-strain value of 0.55 eV by $\approx 45$% and 32% for “Eq–Eq” (Fig. 6d) and “Eq-Ap” (Fig. 6b), respectively. These results appear to be in agreement with the scarce experimental data that is available for compressive biaxial strains^37^ but in apparent disagreement with previous DFT results reported by Al-Hamadany *et al.*^38^. The reasons for the disagreements between our theoretical NEB $E_{a}$ estimations and analogous computational results previously reported^38^ could be the use of different exchange-correlation energy functionals and simulation supercells, which are known to affect the description of polar and antiferrodistortive oxygen octahedral distortions in functional oxide perovskites^47,79^. In order to fully test the reliability of our $E_{a}$ zero-temperature NEB results, we performed complementary *ab initio* molecular dynamics (AIMD) simulations in which lattice thermal excitations are fully taken into account and no particular ${\mathrm{V}_{{\mathrm{O}}}}$ diffusion path needs to be guessed^59^.

### Oxygen ionic diffusion at finite temperatures

Figure 7 encloses the MSD and $D_{\mathrm{O}}$ results obtained from our $T \ne 0$ AIMD simulations for (001) STO thin films at $\eta = \pm 3.6$% and zero strain ("Ab initio molecular dynamics simulations" section). For the $\eta = 0$ case, we estimate large diffusion coefficients of $\sim 10^{-8}$–$10^{-7}$ cm$^{2}$s$^{-1}$ at temperatures higher than 1000 K and a small ${\mathrm{V}_{{\mathrm{O}}}}$ diffusion energy barrier of 0.30 eV (Fig. 7b). The pre-exponential factor entering the corresponding $D_{\mathrm{O}}$ Arrhenius formula ("Ab initio molecular dynamics simulations" section) amounts to $1.8 \cdot 10^{-6}$ cm$^{2}$s$^{-1}$. It is important to note that the $E_{a}$ estimated by fully taking into account lattice thermal excitations is approximately 50% smaller than the corresponding value calculated with the NEB method by considering zero-temperature conditions. This computational outcome demonstrates the existence of an important interplay between lattice vibrations and ${\mathrm{V}_{{\mathrm{O}}}}$ diffusion, which in the case of STO thin films enormously facilitates ionic transport.

It is also worth noting that the agreement between our zero-strain $E_{a}$ result obtained from AIMD simulations and the experimental diffusion energy barrier $E_{a}^{\mathrm{expt}} \approx 0.60$ eV^78^ has considerably worsened as compared to the corresponding NEB estimation. Possible causes explaining such a disagreement could be the neglection of other types of crystalline defects that are relevant to ionic diffusion in our $T \ne 0$ calculations (e.g., dislocations^80^), and the fact that the concentration of oxygen vacancies in our AIMD simulations ($\approx 1.6$%) probably is higher than in the samples that were analyzed in the experiments.

For a tensile strain of $+3.6$%, we find that the diffusion of oxygen ions is considerably enhanced as compared to the $\eta = 0$ case. In particular, we estimate high-*T* diffusion coefficients of $\sim 10^{-7}$ cm$^{2}$s$^{-1}$ and a reduced O diffusion energy barrier of 0.17 eV (Fig. 7b). The value of the pre-exponential factor entering the corresponding $D_{\mathrm{O}}$ Arrhenius formula ("Ab initio molecular dynamics simulations" section) is equal to $2.2 \cdot 10^{-6}$ cm$^{2}$s$^{-1}$. The $E_{a}$ decrease induced by $\eta = +3.6$% is about 50% of the zero-strain value, which is very similar to the relative variation estimated with NEB techniques for the same biaxial strain and “Eq–Eq” vacancy diffusion path ("Zero-temperature activation energy for oxygen diffusion" section). In this case, it is also concluded that the effects of lattice thermal excitations is to significantly enhance oxygen transport. As regards compressive biaxial strains, it is found that even at temperatures as high as 1500 and 2000 K the diffusion coefficient of oxygen atoms is nominally zero (Fig. 7a). This AIMD result is in qualitative agreement with the NEB calculations presented in the previous section, since in the latter case we found that $E_{a}$ increases almost linearly with $|\eta |$ ("Zero-temperature activation energy for oxygen diffusion" section).

Overall, the AIMD simulation results presented in this section confirm the correctness (at the qualitative level) of our NEB results reported in "Zero-temperature activation energy for oxygen diffusion" section, and demonstrate that lattice thermal vibrations have a significant enhancing effect on the diffusion of oxygen ions in (001) STO thin films. Interestingly, it is not always the case that lattice thermal excitations are found to promote ionic transport. For instance, in a recent systematic theoretical study on Li-based fast-ion conductors^59^ the opposite trend has been demonstrated, namely, the energy barriers for ionic transport estimated from AIMD simulations in general are higher than those obtained with NEB methods. It is likely that the degree of anharmonicity of the non-diffusing lattice in the considered material, which determines the amplitude of the atomic fluctuations around the corresponding equilibrium positions, is directly related to the either enhancing or suppressing ionic diffusion effect mediated by the lattice excitations. Further quantitative investigations on this subject deserve future work.

## Conclusions

We have presented a comprehensive *ab initio* study on the formation energy of oxygen vacancies and ionic diffusion properties of epitaxially strained (001) STO thin films, a class of functional materials with great fundamental and applied interests. The novelty of our work lies in the incorporation of lattice thermal excitations on the first-principles description of oxygen vacancies under varying epitaxial strain conditions. It has been demonstrated that in order to achieve an improved agreement with the experimental observations it is necessary to explicitly consider temperature-induced ${\mathrm{V}_{{\mathrm{O}}}}$ lattice effects in the theoretical calculations. By performing quasi-harmonic Gibbs free energy calculations, we have been able to qualitatively reproduce the nonmonotonic peak-like dependence of the ${\mathrm{V}_{{\mathrm{O}}}}$ formation enthalpy measured in STO thin films. Also, by performing *ab initio* molecular dynamics simulations we have been able to reproduce the qualitative $\eta$-driven oxygen ion diffusion trends observed in biaxially strained (001) STO samples. Generalization of our main conclusions to other technologically relevant oxide perovskite materials is likely although further experimental and computational works on the interplay between oxygen vacancies and epitaxial strain are necessary. We hope that the present study will stimulate new research efforts in this direction.

## Supplementary Information

Supplementary information

## Data Availability

The data that support the findings of this study are available from the corresponding author (C.C.) upon reasonable request.
