# Supplementary material for: Ab initio description of oxygen vacancies in epitaxially strained SrTiO3 at finite temperatures
Source: Sci Rep. 2021 Jun 1;11:11499. doi: 10.1038/s41598-021-91018-4 (PMC8169953; doi:10.1038/s41598-021-91018-4)
Supplement: Supplementary file 1 — Supplementary information [file 41598_2021_91018_MOESM1_ESM.pdf]

SUPPLEMENTARY FIGURES

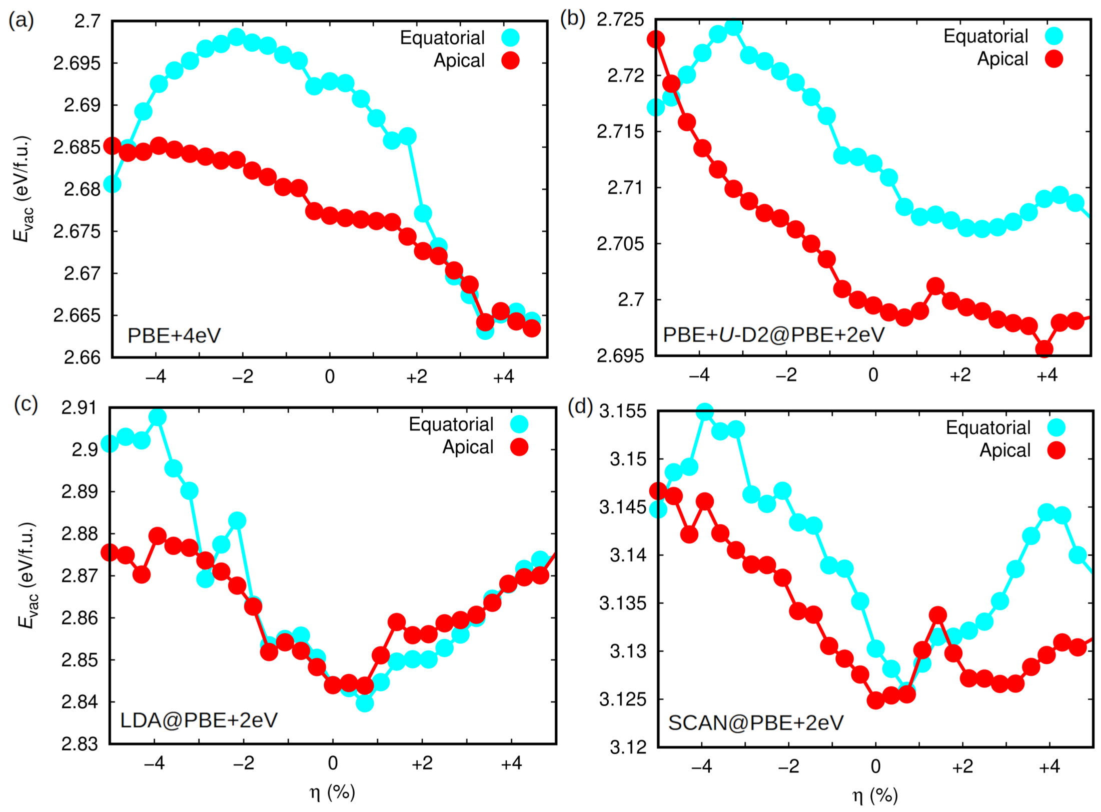

Supplementary Figure 1: Effect of the employed density functional theory (DFT) exchange-correlation energy functional on the calculated formation energy of oxygen vacancies for non-stoichiometric $SrTiO_{2.75}$. (a) PBE+U results obtained by considering $U = 4$ eV [1,2]. (b) PBE+U-D2 results obtained by considering $U = 2$ eV and Grimme D2 dispersion corrections [3]; the Grimme D2 dispersion correction coefficients adopted for Sr and Ti ions are those of Kr and Ar (i.e., closest noble gas elements in the periodic table), respectively, as this computational strategy has been successfully applied to other inorganic crystals in previous works [4,5]. Results obtained for (c) the local density approximation (LDA [6]) and (d) the recently proposed meta-GGA functional SCAN [7,8]. Geometry relaxations were performed only for the PBE+U ($U = 4$ eV) case. All E_{vac} results are qualitatively analogous to those reported for the PBE+U ($U = 2$ eV) functional in the main text.

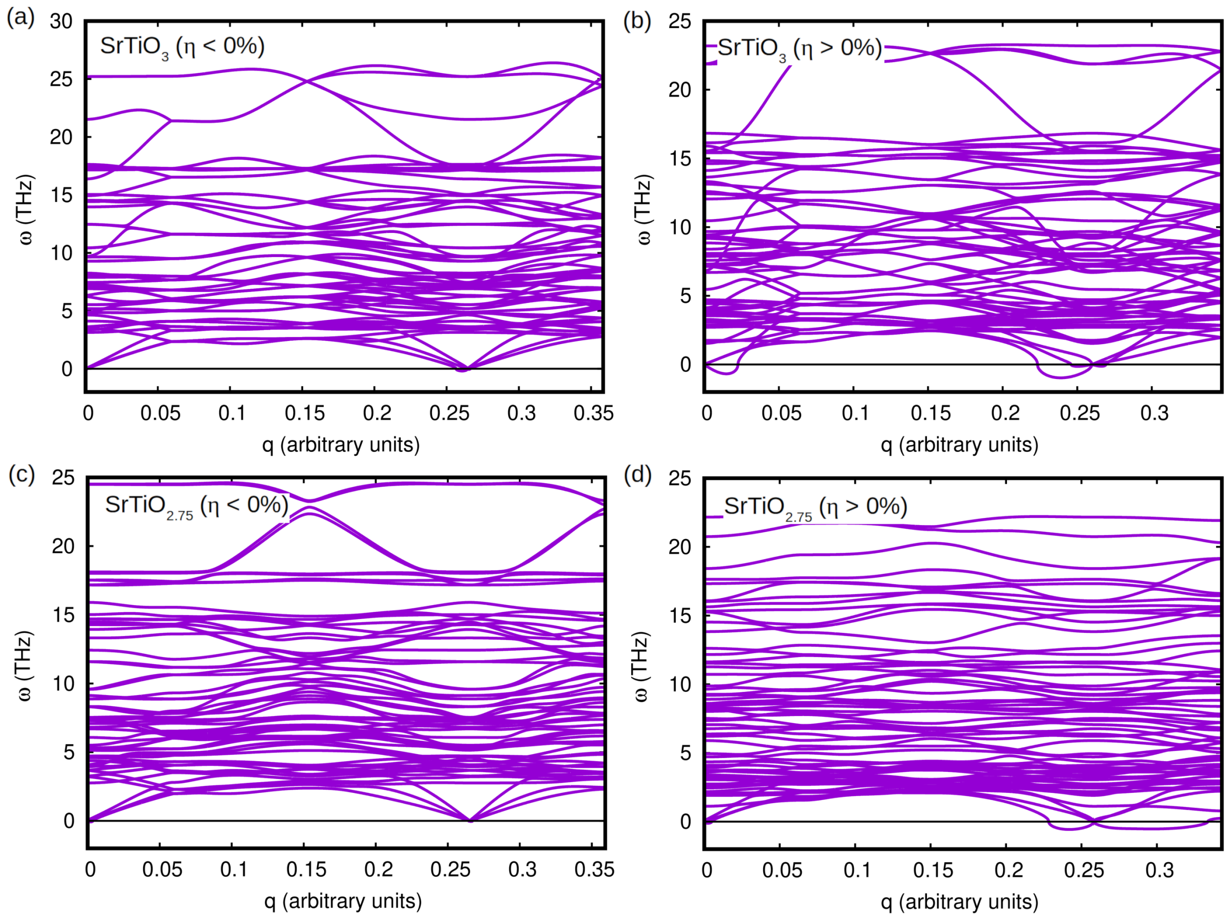

Supplementary Figure 2: Full phonon spectra estimated for stoichiometric and non-stoichiometric STO thin films subjected to large biaxial strains. (a) $SrTiO_3$ under a compressive biaxial strain of -4%. (b) $SrTiO_3$ under a tensile biaxial strain of $+4\%$. (c) $SrTiO_{2.75}$ under a compressive biaxial strain of -4%. (d) $SrTiO_{2.75}$ under a tensile biaxial strain of $+4\%$. For large biaxial tensile strains, small numerical artifacts, probably related to the neglection of long-range quadrupolar and higher-order multipolar interactions between atoms [9], affect the estimation of the acoustic phonon branches at reciprocal space points close to the first Brillouin zone center. Nevertheless, the contribution of such ill-defined phonon frequencies to the quasi-harmonic free energy of STO can be safely neglected (i.e., are disregarded for the evaluation of Eq.(3) in the main text) due to the relatively high temperatures considered in this work (i.e., $500 \leq T \leq 1000$ K) [9,10].

SUPPLEMENTARY REFERENCES

[1] J. P. Perdew, K. Burke, and M. Ernzerhof, Phys. Rev. Lett. **77**, 3865 (1996). Generalized gradient approximation made simple.

[2] S. L. Dudarev, G. A. Botton, S. Y. Savrasov, C. J. Humphreys, and A. P. Sutton, Phys. Rev. B **57**, 1505 (1998). Electron-energy-loss spectra and the structural stability of nickel oxide: An LSDA+U study.

[3] S. Grimme, J. Comput. Chem. **27**, 1787 (2006). Semiempirical GGA-type density functional constructed with a long-range dispersion correction.

[4] M. Cutini, B. Civalleri, and P. Ugliengo, ACS Omega **4**, 1838 (2019). Cost-effective quantum mechanical approach for predicting thermodynamic and mechanical stability of pure-silica zeolites.

[5] E. I. Román-Román and C. M. Zicovich-Wilson, Chem. Phys. Lett. **619**, 109 (2015). The role of long-range van der Waals forces in the relative stability of SiO_2-zeolites.

[6] D. M. Ceperley and B. J. Alder, Phys. Rev. Lett. **45**, 566 (1980). Ground state of the electron gas by a stochastic method.

[7] J. Sun, A. Ruzsinszky, and J. P. Perdew, Phys. Rev. Lett. **115**, 036402 (2015). Strongly constrained and appropriately normed semilocal density functional.

[8] J. Sun, R. Remsing, Y. Zhang *et al.* Nat. Chem. **8**, 831 (2016). Accurate first-principles structures and energies of diversely bonded systems from an efficient density functional.

[9] M. Royo, K. R. Hahn, and M. Stengel, Phys. Rev. Lett. **125**, 217602 (2020). Using high multipolar orders to reconstruct the sound velocity in piezoelectrics from lattice dynamics.

[10] C. Cazorla and T. Gould, Sci. Adv. **5**, eaau5832 (2019). Polymorphism of bulk boron nitride.
